# Supplementary material for: A large scale hearing loss screen reveals an extensive unexplored genetic landscape for auditory dysfunction
Source: Nat Commun. 2017 Oct 12;8:886. doi: 10.1038/s41467-017-00595-4 (PMC5638796; doi:10.1038/s41467-017-00595-4)
Supplement: Supplementary file 1 — Supplementary Information [file 41467_2017_595_MOESM1_ESM.pdf]

SI GUIDE

File Name: Supplementary Information
Description: Supplementary Tables.

File Name: Supplementary Data
Description: Initial candidate hearing loss lines.

File Name: Peer Review File
Description:

Supplementary Table 1. Sixty-seven Hearing Loss Genes Identified from the IMPC programme

Gene	Symbol	Allele	Zygosity	Screened	Centre	Status	HL
RIKEN cDNA A730017C20 gene	*A730017C20Rik*	A730017C20Rik$^{tm1b(KOMP)Wtsi}$	Hom	M+F	WTSI	Novel	Severe
AP2 associated kinase 1	*Aak1*	Aak1$^{tm1b(EUCOMM)Hmgu}$	Hom	M+F	ICS	Novel	High
acyl-CoA synthetase long-chain family member 4	*Acsl4*	Acsl4$^{tm1a(EUCOMM)Wtsi}$	Hom	F	WTSI	Novel	High
activin receptor IIA	*Acvr2a*	Acvr2a$^{tm1.1(KOMP)Vlcg}$	Hom	F	TCP	Novel	Mild
adhesion G protein-coupled receptor B1	*Adgrb1*	Adgrb1$^{tm2a(EUCOMM)Wtsi}$	Hom	M+F	WTSI	Novel	Mild
adhesion G protein-coupled receptor V1	*Adgrv1*	Adgrv1$^{tm1.1(KOMP)Vlcg}$	Hom	M+F	UCD	Known	Severe
alpha-2-HS-glycoprotein	*Ahsg*	Ahsg$^{tm1.1(KOMP)Vlcg}$	Hom	M+F	UCD	Novel	High
ankyrin repeat domain 11	*Ankrd11*	Ankrd11$^{tm1b(EUCOMM)Wtsi}$	Het	M+F	ICS	Novel	Mild
adaptor-related protein complex 3, mu 2 subunit	*Ap3m2*	Ap3m2^{em1H}	Hom	M+F	H	Novel	Mild
adaptor-related protein complex 3, sigma 1 subunit	*Ap3s1*	Ap3s1$^{tm1b(EUCOMM)Hmgu}$	Hom	M+F	GMC	Novel	Mild
ATPase, Ca++ transporting, plasma membrane 1	*Atp2b1*	Atp2b1$^{tm1b(KOMP)Wtsi}$	Het	M+F	UCD	Novel	Low
RIKEN cDNA B020004J07	*B020004J07Rik*	B020004J07Rik$^{tm1b(KOMP)Wtsi}$	Hom	M+F	UCD	Novel	Low
BAI1-associated protein 2-like 2	*Baiap2l2*	Baiap2l2$^{tm1b(EUCOMM)Hmgu}$	Hom	M+F	ICS	Novel	Mild
coiled-coil domain containing 88C	*Ccdc88c*	Ccdc88c$^{tm1b(KOMP)Mbp}$	Hom	M+F	UCD	Novel	High
coiled-coil domain containing 92	*Ccdc92*	Ccdc92$^{tm1b(KOMP)Mbp}$	Hom	M+F	UCD	Novel	Mild
calcium and integrin binding family member 2	*Cib2*	Cib2$^{tm1b(EUCOMM)Wtsi}$	Hom	M+F	H	Known	Severe
clarin 1	*Clrn1*	Clrn1$^{tm1.1(KOMP)Vlcg}$	Hom	M+F	UCD	Known	Severe
collagen, type IX, alpha 2	*Col9a2*	Col9a2$^{tm1b(EUCOMM)Wtsi}$	Hom	M+F	H	Known	Severe
cytochrome b5 reductase 2	*Cyb5r2*	Cyb5r2$^{tm1b(EUCOMM)Hmgu}$	Hom	M+F	H	Novel	Mild
deoxyribonucleas e I	*Dnase1*	Dnase1$^{tm1.1(KOMP)Vlcg}$	Hom	M+F	UCD	Novel	High
dual oxidase maturation factor 2	*Duoxa2*	Duoxa2$^{tm1b(KOMP)Wtsi}$	Hom	M+F	WTSI	Novel	Severe
ELMO/CED-12 domain containing 1	*Elmod1*	Elmod1$^{tm1b(EUCOMM)Hmgu}$	Hom	M+F	H	Known	Severe
embigin	*Emb*	Emb$^{tm1b(KOMP)Wtsi}$	Hom	F	UCD	Novel	High
EPS8-like 1	*Eps8l1*	Eps8l1$^{tm2b(KOMP)Wtsi}$	Hom	M+F	UCD	Novel	Severe
Ewing sarcoma breakpoint region 1	*Ewsr1*	Ewsr1$^{tm1b(EUCOMM)Wtsi}$	Het	F	TCP	Novel	High
GATA binding protein 2	*Gata2*	Gata2$^{tm1b(KOMP)Wtsi}$	Het	M+F	UCD	Known	Low
golgi associated, gamma adaptin ear containing, ARF binding protein 1	*Gga1*	Gga1$^{tm1.1(KOMP)Vlcg}$	Hom	M+F	UCD	Novel	Mild
GIPC PDZ domain containing family, member 3	*Gipc3*	Gipc3$^{tm1b(KOMP)Wtsi}$	Hom	M+F	JAX	Known	Severe
G protein-coupled receptor 152	*Gpr152*	Gpr152$^{tm1b(EUCOMM)Wtsi}$	Hom	M+F	WTSI	Novel	High
G-protein-coupled	*Gpr50*	Gpr50$^{tm1.1(KOMP)Vlcg}$	Hom	F	UCD	Novel	Low

receptor 50							
IKAROS family zinc finger 5	*Ikzf5*	Ikzf5$^{tm1(KOMP)Wtsi}$	Hom	M	RBRC	Novel	High
interleukin 1 receptor, type II	*Il1r2*	Il1r2$^{tm1.1(KOMP)Vlcg}$	Hom	M+F	UCD	Novel	Low
immunoglobulin-like domain containing receptor 1	*Ildr1*	Ildr1$^{tm1(KOMP)Wtsi}$	Hom	M+F	WTSI	Known	Severe
kinesin light chain 2	*Klc2*	Klc2$^{tm1e(EUCOMM)Wtsi}$	Hom	M+F	WTSI	Novel	Severe
kelch-like 18	*Klhl18*	Klhl18$^{tm1a(KOMP)Wtsi}$	Hom	M+F	WTSI	Novel	Low
MARVEL (membrane-associating) domain containing 2	*Marveld2*	Marveld2$^{tm1b(EUCOMM)Wtsi}$	Hom	M+F	H	Known	Severe
mediator complex subunit 28	*Med28*	Med28$^{tm1.1(KOMP)Vlcg}$	Het	M+F	UCD	Novel	Low
multiple PDZ domain protein	*Mpdz*	Mpdz^{em1J}	Hom	M+F	JAX	Novel	Mild
myosin, heavy polypeptide 1, skeletal muscle, adult	*Myh1*	Myh1$^{tm1b(KOMP)Wtsi}$	Hom	M+F	JAX	Novel	Mild
myosin VIIA	*Myo7a*	Myo7a$^{tm1a(EUCOMM)Wtsi}$	Hom	M+F	WTSI	Known	Severe
neural precursor cell expressed, developmentally down-regulated gene 4-like	*Nedd4l*	Nedd4l$^{tm1b(KOMP)Wtsi}$	Hom	M+F	H	Novel	Severe
nuclear factor of activated T cells, cytoplasmic, calcineurin dependent 3	*Nfatc3*	Nfatc3$^{tm1b(EUCOMM)Hmgu}$	Het	M+F	GMC	Novel	Low
ninein	*Nin*	Nin$^{tm1b(EUCOMM)Hmgu}$	Hom	M	RBRC	Novel	High
nischarin	*Nisch*	Nisch$^{tm1b(EUCOMM)Hmgu}$	Hom	M+F	H	Novel	Mild
neuroplastin	*Nptn*	Nptn$^{tm1b(EUCOMM)Hmgu}$	Hom	M+F	H	Novel	Severe
oncomodulin	*Ocm*	Ocm$^{tm1e(EUCOMM)Wtsi}$	Hom	M+F	WTSI	Known	Severe
outer dense fiber of sperm tails 3-like 2	*Odf3l2*	Odf3l2$^{tm1.1(KOMP)Vlcg}$	Hom	M+F	UCD	Novel	Mild
otoancorin	*Otoa*	Otoa$^{tm1.1(KOMP)Vlcg}$	Hom	M+F	UCD	Known	Severe
PHD finger protein 6	*Phf6*	Phf6$^{tm1b(EUCOMM)Wtsi}$	Het	F	ICS	Novel	High
protein phosphatase 1A, magnesium dependent, alpha isoform	*Ppm1a*	Ppm1a$^{tm1b(EUCOMM)Hmgu}$	Hom	M+F	H	Novel	High
sema domain, immunoglobulin domain (Ig), short basic domain, secreted, (semaphorin) 3F	*Sema3f*	Sema3f$^{tm1b(EUCOMM)Hmgu}$	Hom	M+F	H	Novel	Low
solute carrier family 4, sodium bicarbonate cotransporter-like, member 10	*Slc4a10*	Slc4a10$^{tm1b(KOMP)Wtsi}$	Hom	M+F	H	Novel	Mild
solute carrier family 5 (sodium iodide symporter), member 5	*Slc5a5*	Slc5a5$^{tm1b(KOMP)Wtsi}$	Hom	M+F	JAX	Novel	Severe
spinster homolog 2	*Spns2*	Spns2$^{tm1b(KOMP)Wtsi}$	Hom	M+F	WTSI	Novel	Severe
serine/arginine repetitive matrix 4	*Srrm4*	Srrm4$^{tm1e(EUCOMM)Wtsi}$	Hom	M+F	WTSI	Known	Mild
transmembrane protein 30B	*Tmem30b*	Tmem30b$^{tm1b(EUCOMM)Wtsi}$	Hom	M+F	UCD	Novel	Severe
transmembrane and tetratricopeptide repeat containing 4	*Tmtc4*	Tmtc4$^{tm1.1(KOMP)Vlcg}$	Hom	M+F	UCD	Novel	Severe
thymocyte	*Tox*	Tox$^{tm1b(KOMP)Wtsi}$	Hom	M+F	UCD	Novel	Severe

selection-associated high mobility group box							
taperin	*Tprn*	$\mathrm{Tprn}^{\mathrm{tm1.1(KOMP)Vlcg}}$	Hom	M+F	JAX	Known	Severe
translocating chain-associating membrane protein 2	*Tram2*	$\mathrm{Tram2}^{\mathrm{tm1a(KOMP)Wtsi}}$	Hom	M+F	WTSI	Novel	Mild
ubiquitin-conjugating enzyme E2B	*Ube2b*	$\mathrm{Ube2b}^{\mathrm{tm1b(EUCOMM)Wtsi}}$	Het	M+F	H	Novel	Mild
ubiquitin-conjugating enzyme E2G 1	*Ube2g1*	$\mathrm{Ube2g1}^{\mathrm{tm1b(EUCOMM)Hmgu}}$	Hom	M	RBRC	Novel	Mild
Usher syndrome 1C	*Ush1c*	$\mathrm{Ush1c}^{\mathrm{tm1a(KOMP)Wtsi}}$	Hom	M+F	WTSI	Known	Severe
vesicle transport through interaction with t-SNAREs 1A	*Vti1a*	$\mathrm{Vti1a}^{\mathrm{tm1.1(KOMP)Vlcg}}$	Hom	M+F	UCD	Novel	Mild
WD and tetratricopeptide repeats 1	*Wdtc1*	$\mathrm{Wdtc1}^{\mathrm{tm1a(KOMP)Wtsi}}$	Hom	M+F	WTSI	Novel	High
zinc finger, CCHC domain containing 14	*Zcchc14*	$\mathrm{Zcchc14}^{\mathrm{tm1a(KOMP)Wtsi}}$	Hom	M+F	WTSI	Novel	Low
zinc finger protein 719	*Zfp719*	$\mathrm{Zfp719}^{\mathrm{tm1b(EUCOMM)Wtsi}}$	Hom	M+F	WTSI	Novel	Severe

For each of the 67 mutant strains exhibiting a hearing loss phenotype the following information is provided: Full gene name; Gene symbol; Allele tested; Zygosity of animals tested, homozygous (Hom) or heterozygous (Het); Sex of mice tested, males only (M), females only (F) or both (M+F); Phenotyping Centre, Harwell (H), Helmholtz Zentrum Munchen (GMC), Institut Clinique de la Souris (ICS), Jackson Laboratories (JAX), RIKEN Tsukuba Institute, BioResource Center (RBRC), The Centre for Phenogenomics (TCP), University of California, Davis (UCD), Wellcome Trust Sanger Institute (WTSI); Hearing loss gene status; and, Type of hearing loss.

Supplementary Table 2: Nine Hearing Loss-Associated Genes Not Showing an Auditory Phenotype in IMPC. Note that for six of these genes hearing loss is reported only in human patients. For the gene, *Myo3a*, the hearing loss in the reported homozygous mouse mutant is progressive and mild and might not have been detected in the IMPC screen. Similarly, for *P3h1*, hearing loss in the reported homozygous mouse mutant is mild. *Eya4* mutant heterozygotes were screened in IMPC and, though *Eya4* homozygotes show raised thresholds due to the development of otitis media, no data is available on auditory thresholds for *Eya4* heterozygote mice.

Gene	Symbol	Allele	Zygosity	Screened	Deafness-association	Refs
Activin A receptor, type 1	*Acvr1*	$Acvr1^{tm1.1(KOMP)Vlcg}$	Het	M+F	Fibrodysplasia Ossificans Progressiva (FOP; #135100) is caused by heterozygous mutation in the *ACVR1* gene (#102576). To investigate the clinical features of this condition, Morales-Piga et al. evaluated a cohort of Spanish FOP patients. Of these, 7 of 18 (38.9%) reported some hearing loss.	Morales-Piga, A. *et al*. (2012). *Bone*. **51**:748-55
Crystallin, mu	*Crym*	$Crym^{tm1b(KOMP)Wtsi}$	Hom	M+F	Heterozygous mutation in the *CRYM* gene (#123740) has been reported by Abe *et al* to cause autosomal dominant deafness, DFNA40 (#616357), in two separate Japanese families. To investigate the *in vivo* functions of CRYM, Suzuki *et al* generated mice with targeted disruption of the *Crym* gene (*Crym*$^{-}$). Auditory assessment of these mice found that hearing thresholds of *Crym*$^{-/-}$ were not significantly different from those of *Crym*$^{+/+}$ mice.	Abe, S. *et al*. (2003). *American Journal of Human Genetics*. **72**:73-82 Suzuki, S. *et al*. (2007). *Molecular Endocrinology*. **21**:885-94
Dentin sialophosphoprotein	*Dspp*	$Dspp^{tm1.1(KOMP)Vlcg}$	Hom	M+F	Dentinogenesis imperfecta 1 (DGI1, #125490) is an autosomal dominant dental disease characterized by abnormal dentin production and mineralization and is caused by mutations in the *DSPP* gene (#125485). Xiao *et al* report three Chinese families with DGI1 due to different *DSPP* mutations, and affected individuals from two of these families also presented with progressive sensorineural high-frequency hearing loss (DFNA39).	Xiao, S. *et al*. (2001). *Nature Genetics*. **27**:201-4
EYA transcriptional coactivator and phosphatase 4	*Eya4*	$Eya4^{tm1b(KOMP)Wtsi}$	Het	M+F	Autosomal dominant deafness, DFNA10 (#601316), is caused by heterozygous mutation in the *EYA4* gene (#603550). Wayne et al reported different missense mutations in two separate families segregating with postlingual, progressive, autosomal dominant hearing loss. Depreux et al generated and characterized Eya4-deficient (*Eya4*$^{-/-}$) mice. These had severe hearing deficits and developed otitis media with effusion (no auditory threshold data is provided for heterozygous (*Eya4*$^{+/-}$) mice).	Wayne, S. *et al*. (2001). *Human Molecular Genetics*. **10**:195-200 Depreux, FS. *et al*. (2008). *Journal of Clinical Investigation*. **118**:651-658
Gap junction protein, beta 3	*Gjb3*	$Gjb3^{tm1.1(KOMP)Vlcg}$	Het	M+F	Autosomal dominant deafness, DFNA2B (#612644), is caused by mutation in the *GJB3* gene (#603324), which encodes Connexin 31 (Cx31). To study the function of this gene in mice, Plum et al. generated a Cx31-deficient model (*Gjb3*$^{-/-}$). While embryonic lethality was observed in null mice, some survived to adulthood. No morphological or functional inner ear defects were observed in the surviving *Gjb3*$^{-/-}$ mice, suggesting Cx31-deficiency is compensated for by other connexins.	Plum, A. *et al*. (2001). *Developmental Biology*. **231**:334-47
G-protein signalling modulator 2	*Gpsm2*	$Gpsm2^{tm1b(EUCOMM)Wtsi}$	Het	M+F	Chudley-McCullough syndrome (CMCS; #604213) is caused by homozygous or compound heterozygous mutations in the *GPSM2* gene (#609245). CMCS is an autosomal recessive neurologic disorder characterized by early-onset sensorineural deafness and specific brain anomalies on MRI. Bhonker et al utilized a targeted deletion line to assess the requirement of *Gpsm2* for hearing in the mouse. They found that while homozygous mutants were profoundly deaf, heterozygous mice had hearing thresholds that were similar to wild-type controls.	Bhonker, Y. *et al*. (2016). *Mammalian Genome*. **27**:29-46
Myosin IIIA	*Myo3a*	$Myo3a^{tm1b(KOMP)Wtsi}$	Hom	M+F	Autosomal recessive deafness, DFNB30 (#607101), is caused by mutations in the *MYO3A* gene (#606808). Walsh *et al* generated a mouse model (*Myo3a*$^{KI/KI}$) that harbors a mutation equivalent to the nonsense allele responsible for the most severe human DFNB30 phenotype. While auditory function in these mice is impaired and progressively deteriorates with age, ABR thresholds measured at 2.5 months of age were only mildly elevated at 6 to 30 kHz.	Walsh, VL. *et al*. (2011). *Mammalian Genome*. **22**:170-177
Snail family zinc finger 2	*Snai2*	$Snai2^{tm1.1(KOMP)Vlcg}$	Hom	F	Waardenburg syndrome type 2D (#608890) is caused by homozygous deletion of the *SNAI2* gene (#602150), and characterized by pigmentary abnormalities of the hair, skin, and eyes, and congenital sensorineural hearing loss. Sánchez-Martín et	Sánchez-Martín, M. *et al*. (2002). *Human Molecular Genetics*. **11**:3231-3236

					al. investigated mice lacking Snai2 and found them to have patchy deficiency of melanocytes, a phenotype similar to that observed in patients with Waardenburg syndrome. Hearing function was not assessed in the *Snai2*$^{-/-}$ mice, but hyperactivity and circling in some mutants was observed suggesting a possible inner ear defect.	
Prolyl 3-hydroxylase 1	*P3h1*	P3h1$^{tm1b(EUCOMM)Wtsi}$	Hom	M+F	Autosomal recessive osteogenesis imperfecta type VIII (OI8; #610915) is caused by homozygous or compound heterozygous mutation in the *Ph31* gene (#610339). While deafness is not reported in the clinical synopsis of OI8, hearing loss is a common problem for people with osteogenesis imperfecta. Pokidysheva *et al* report that *P3h1* null mice exhibit mild hearing impairment and abnormal morphology of the middle ear bone joints	Pokidysheva, E. *et al*. (2013). *Matrix Biology*. **32**:39-44

For each of the 9 discounted lines that have association with hearing loss the following information is provided: Full gene name; Gene symbol; Allele tested; Zygosity of animals tested, homozygous (Hom) or heterozygous (Het); Gender of mice tested, females only (F) or males and females (M+F); Details of the associated deafness phenotype, including Phenotype and Gene MIM numbers.

Supplementary Table 3. Collated data showing the procedural phenotyping results for each of the 67 hearing loss-associated candidate genes.

**Hearing loss classification** Gene	Zygosity of tested mutants	Procedural phenotyping data: Body Composition	Calorimetry	Clinical Blood Chemistry	Eye Morphology	Fertility and Viability	Glucose Tolerance Test	Grip Strength	Heart Function	Hematology	Insulin Blood Level	Open field	Organ Weight	Gross Pathology and Histopathology	X-ray	Combined SHIRPA and Dysmorphology	Total number of significant phenotypes / Total number of parameters tested
Severe																	
A730017C20Rik	Hom	8/9	0/9	2/27	0/26	0/3	0/4	0/6		0/10	0/1		0/1		2/41	1/115	13/252
Adgrv1	Hom	0/9	0/4	0/15	0/9	0/1	0/4	0/6	0/5	0/20	0/1	0/21	0/1		1/25	1/77	2/198
Cib2	Hom	0/8	2/6	1/23	0/27	0/3	0/4	0/6	1/25	0/22		0/25	0/1	0/28	0/27	3/77	7/282
Clrn1	Hom		0/4	0/15	0/9	0/3	0/4	0/6	0/5	0/20	0/1	0/21	0/1	0/29	0/23	2/77	2/218
Col9a2	Hom	0/8	0/6	2/23	0/27	0/3	0/4	0/6	0/14	0/22		0/25	0/1	0/28	8/27	2/77	12/271
Duoxa2	Hom	8/9	0/9	17/27	2/26	0/1	2/4	6/6		10/10	1/1		1/1		10/42	7/115	64/251
Elmod1	Hom	7/8	5/6	3/23	0/27	0/3	0/4	6/6	1/26	6/22		18/25	0/1	0/28	1/26	7/77*	54/282
Eps8l1	Hom	0/9	0/4	0/16	0/9	0/1	0/4	0/6	0/5	0/20	0/1	0/21	0/1	0/29	0/25	0/77	0/228
Gipc3	Hom	0/9		0/19	0/31	0/3	0/4	0/6	0/11	0/8	0/1	0/13	0/1	0/21		0/77	0/204
Ildr1	Hom	0/9	0/8	0/26	0/26	0/3	0/4	0/6		0/10	0/1				0/41	1/115	1/249
Klc2	Hom	0/9	0/8	0/27	0/26	0/3	0/4	0/6		0/10	0/1				0/14	1/115	1/223
Marveld2	Hom	0/8	0/6	0/23	0/27	0/3	0/4	0/6	0/15	0/22		0/25	0/1	0/29	0/27	2/77	2/273
Myo7a	Hom	1/9	0/8	2/27	0/26	0/3	0/4	0/6		0/10	0/1		0/1		0/41	4/115*	7/251
Nedd4l	Hom	0/8	0/6	0/23	1/27	1/3	0/4	0/6	0/15	0/22		4/25	0/1	0/27	0/27	1/77	7/271
Nptn	Hom	4/8	3/6	1/23	0/27	0/1	0/4	0/6	0/26	4/22		9/25	0/1	0/28	0/27	5/77	26/281
Ocm	Hom	0/9	0/8	0/27	0/26	0/3	0/4	0/6		0/10	0/1		0/1		0/41	1/115	1/251
Otoa	Hom	0/9	0/4	0/16	0/9	0/3	0/4	0/6	0/5	0/20	0/1	0/21	0/1	0/29	0/25	0/77	0/230
Slc5a5	Hom	1/9		1/19	0/31	0/3	0/4	0/6	1/11	0/8		1/13	0/1	0/21		0/77	4/203
Spns2	Hom	2/9	0/9	3/26	5/18	0/3	0/4	0/6		2/10	0/1		0/1		0/36	2/115	14/238
Tmem30b	Hom		0/4	0/16	0/9	0/3	0/4	0/6	0/5	0/20	0/1	0/21	0/1	0/29	0/23	3/77	3/219
Tmtc4	Hom		0/4	0/15	0/9	0/2		0/6	0/5	0/20	0/1	0/21	0/1	0/29	0/23	1/77	1/213
Tox	Hom		0/4	2/16	0/9	0/3	0/4	0/6	0/5	0/20	0/1	0/21	0/1	0/29	0/23	1/77	3/219
Tprn	Hom	0/9		0/19	0/31	0/3	0/4	0/6	0/11	0/8	0/1	0/13	0/1	0/21		1/77	1/204
Ush1c	Hom	5/9	0/8	8/27	0/26	0/3	2/4	5/6		1/10	1/1		0/1		1/41	5/115*	27/251
Zfp719	Hom	0/9	0/9	0/26	0/26	0/3	0/4	0/6		0/10	0/1		0/1		0/1	1/115	1/211
Mild																	
Acvr2a	Hom	0/9	0/8	0/18	0/27	1/1	0/4	0/6	0/11	0/14		0/28	0/1	0/25	0/25	0/77	1/254
Adgrb1	Hom	0/9	0/8	2/27	0/26	0/3	0/4	0/6		0/10	0/1		0/1		0/41	0/115	2/251
Ankrd11	Het	2/9	0/10	0/16	0/46		0/4	0/6	0/12	1/20		0/17	0/2	0/29	0/25	1/77	4/273
Ap3m2	Hom	0/8	0/6	0/23	0/27	0/1	0/4	0/6	0/14	0/22		0/25	0/1	0/28	0/28	0/77	0/270
Ap3s1	Hom	0/5		1/24	1/39	0/3	1/4		1/22	0/10			0/2	0/29	0/25		4/163
Baiap2l2	Hom	0/9	0/10	0/16	1/46	0/3	0/4	0/6	0/12	0/20		0/17	0/2	0/29	0/25	1/77	2/276
Ccdc92	Hom		0/4	1/16	0/9	0/3	0/4	2/6	0/5	0/20	0/1	0/21	0/1	0/29	0/23	0/77	3/219
Cyb5r2	Hom	0/8	0/6	0/23	0/27	0/3	0/4	0/6	0/26	0/22		0/25	0/1	0/28	0/28	0/77	0/284
Gga1	Hom	0/9	0/4	0/15	0/9	2/3	0/4	0/6	0/5	0/20	0/1	0/21	0/1	0/29	0/23	0/77	2/227
Mpdz	Hom	4/9		0/19	6/31	0/3	0/4	5/6	0/11	1/8	0/1	0/13	0/1	0/21		0/77	16/204
Myh1	Hom	6/9		0/19	0/31	0/1	0/4	6/6	0/11	4/8		2/13	0/1	0/21		2/77	20/201
Nisch	Hom	6/8	0/6	3/23	0/27	0/1	1/4	2/6	0/14	0/22		2/25	0/1	0/28	0/28	0/77	14/270
Odf3l2	Hom	0/9	0/4	0/15	0/9	0/3	0/4	0/6	0/5	0/20	0/1	0/21	0/1	0/29	0/25	0/77	0/229
Slc4a10	Hom	5/8	0/6	2/23	0/27	0/1	0/4	0/6	0/14	0/22		7/25	0/1	0/28	0/28	1/77	15/270
Srrm4	Hom	0/9	0/8	0/27	0/26	1/3	0/4	0/6		0/10	0/1		0/1		0/41	0/115	1/251
Tram2	Hom	4/9	0/8	2/27	0/26	0/3	0/4	1/6		1/10	1/1		0/1		8/41	1/115	18/251
Ube2b	Het	0/8		0/23	0/27		0/4	0/6	0/26	0/22		0/25	0/1	0/28	0/27	3/77	3/274
Ube2g1	Hom	3/9	0/10	3/22	1/33	1/3	2/4	0/6	1/7	8/22		3/15		0/20	5/40	2/77	29/268
Vti1a	Hom	1/9	0/4	1/15	0/9	0/3	1/4	0/6	1/5	0/20	1/1	1/21	0/1	0/29	0/23	1/77	7/227
High frequency																	
Aak1	Hom	4/9	3/6	5/15	1/44	1/3	0/4	4/6	1/12	1/20		2/17	2/2	0/29	1/25	0/77	25/270
Acsl4	Hom	0/9		0/27	0/26	1/3	0/4	0/6		0/10	0/1		0/1		0/36	0/115	1/238
Ahsg	Hom	0/9	0/4	0/16	1/9	0/1	0/4	0/6	0/5	0/20	0/1	0/21	0/1	0/29	1/25	0/77	2/228
Ccdc88c	Hom	2/9	0/4	0/15	0/9	0/3	1/4	0/6	0/5	0/20	0/1	0/21	0/1	0/28		0/77	3/203
Dnase1	Hom		0/4	0/16	0/9	0/3		0/6	0/5	0/20	1/1	0/21	0/1	0/29		0/77	1/192
Emb	Hom		0/4	0/16	0/9	1/1	0/4	0/6	0/5	0/20	0/1	0/21	0/1	0/25	0/25	0/77	1/215
Ewsr1	Het	2/9	3/8	1/18	0/27		0/4	0/6	0/11	0/15		2/28	0/1	0/29	1/25	0/77	9/258
Gpr152	Hom	1/9	0/9	1/27	0/26	0/3	0/4	5/6		0/10	0/1		0/1		0/41	1/115	8/252
Ikzf5	Hom	1/9		1/22	0/33	0/3	0/4	0/6	0/7	0/22			0/1	0/20	0/40	0/77	2/244
Nin	Hom	0/9	0/10	0/22	0/33	0/3	0/4	0/6	1/7	0/22		0/15	0/1	0/20	0/40	0/77	1/269
Phf6	Het	0/9		0/16	0/46		0/4	5/6	0/12	0/20		0/17	0/2	0/29	0/25	0/77	5/263
Ppm1a	Hom	1/8	0/6	0/23	0/27	0/3	1/4	0/6	0/15	0/22		0/25	0/1	0/28	0/27	0/77	2/272
Wdtc1	Hom	0/9		3/27	0/26	0/3	0/4	0/6		0/10	0/1		0/1		1/41	0/115	4/243
Low frequency																	
Atp2b1	Het	0/9	3/4	0/15	0/9		0/4	0/6	0/5	0/20		0/21	0/1	0/29	1/25	2/77	6/225
B020004J07Rik	Hom	0/9	0/4	0/14	0/9	0/3	0/4	0/6	0/5	0/20	0/1	0/21	0/1	0/29	0/25	1/77	1/228
Gata2	Het	0/9	0/4	1/15	0/9		0/4	0/6	0/5	0/20	0/1	0/21	0/1	0/29	0/25	0/77	1/226
Gpr50	Hom	0/9		0/15	0/9	0/3	0/4	0/6	0/5		0/1	0/21	0/1	0/25	1/25	0/77	1/201
Il1r2	Hom	0/9	0/4	1/16	0/9	0/3	0/4	0/6	0/5	0/20	0/1	0/21	0/1	0/29	0/25	0/77	1/230
Klhl18	Hom	0/9	0/8	0/27	0/26	0/3	0/4	4/6		0/10	0/1				0/41	0/115	4/250
Med28	Het	0/9	0/4	0/16	0/9		0/4	0/6	0/5	0/20	0/1	0/21	0/1	0/29		0/77	0/202
Nfatc3	Het	0/5	0/5	0/24	5/39		0/4	0/6	0/22	0/10	0/1	0/17	0/2	0/29	0/25	0/77	5/266
Sema3f	Hom	5/8	0/6	6/23	1/27	1/3	0/4	0/6		0/22		0/25	0/1	0/28	1/27	6/77	20/257
Zcchc14	Hom	1/9	0/8	0/27	0/26	0/2	0/4	0/6		0/10	0/1		0/1		0/41	0/115	1/250

The majority of mutants (60 of the 67) display additional phenotypes, while only 7 (~10%) do not show phenodeviance for any other parameter tested. *P* value threshold <0.0001. Red, Phenodeviance detected; Blue, Phenodeviance detected including absent startle response; White, No phenodeviance detected; Grey, No data. White asterisk denotes a head bobbing/circling phenodeviance. For each mutant and procedure the number of significant phenotypes/number of parameters tested are given. All primary data are available on the IMPC portal.

Supplementary Table 4. Gene Ontology Analysis of known and novel hearing loss genes

		KNOWN AND NOVEL DEAFNESS GENES		KNOWN DEAFNESS GENES		NOVEL DEAFNESS GENES	
**GO Categories**	**GO Term**	**Corrected p-value**	**# Genes**	**Corrected p-value**	**# Genes**	**Corrected p-value**	**# Genes**
BP	sensory perception of sound	7.18E-78	52	6.77E-90	52	NA	NA
BP	sensory perception of mechanical stimulus	5.61E-75	52	5.47E-87	52	NA	NA
BP	inner ear development	1.80E-19	23	8.54E-24	23	NA	NA
BP	sensory perception	1.20E-18	52	4.01E-29	52	NA	NA
BP	ear development	4.12E-18	23	2.07E-22	23	NA	NA
BP	neurological system process	2.06E-17	55	2.12E-27	54	1.00E+00	1
BP	system process	2.05E-15	59	3.68E-24	56	1.00E+00	3
BP	ear morphogenesis	7.38E-15	17	5.89E-18	17	NA	NA
BP	inner ear morphogenesis	2.35E-13	15	4.63E-16	15	NA	NA
BP	inner ear receptor cell differentiation	5.43E-13	13	2.53E-15	13	NA	NA
BP	mechanoreceptor differentiation	1.98E-12	13	9.35E-15	13	NA	NA
BP	inner ear receptor cell development	3.06E-11	11	3.45E-13	11	NA	NA
BP	sensory organ development	3.49E-11	25	1.83E-14	24	1.00E+00	1
BP	hair cell differentiation	1.82E-10	10	3.17E-12	10	NA	NA
BP	inner ear receptor stereocilium organization	1.29E-09	9	3.46E-11	9	NA	NA
BP	auditory receptor cell development	1.53E-09	8	6.25E-11	8	NA	NA
BP	auditory receptor cell stereocilium organization	2.05E-09	7	1.28E-10	7	NA	NA
BP	auditory receptor cell differentiation	2.39E-09	9	6.43E-11	9	NA	NA
BP	embryonic organ morphogenesis	3.12E-09	18	2.26E-12	18	NA	NA
BP	sensory organ morphogenesis	4.30E-09	17	4.66E-12	17	NA	NA
BP	multicellular organismal process	6.94E-09	91	1.88E-12	72	1.00E+00	19
BP	auditory receptor cell morphogenesis	1.35E-08	7	8.45E-10	7	NA	NA
BP	embryonic organ development	3.04E-08	20	1.13E-11	20	NA	NA
BP	anatomical structure morphogenesis	1.44E-07	46	7.92E-10	39	1.00E+00	7
BP	neuroepithelial cell differentiation	1.80E-07	9	5.06E-09	9	NA	NA
BP	anatomical structure development	1.92E-07	71	6.46E-10	57	1.00E+00	14
BP	epithelial cell development	5.01E-07	14	2.04E-09	14	NA	NA
BP	cell development	1.13E-06	39	1.16E-07	32	1.00E+00	7
BP	regulation of biological quality	1.40E-06	50	1.18E-04	35	1.00E+00	15
BP	detection of mechanical stimulus involved in sensory perception of sound	1.42E-06	6	1.38E-07	6	NA	NA
BP	single-multicellular organism process	1.52E-06	72	1.94E-07	55	1.00E+00	17
BP	equilibrioception	1.56E-06	5	2.30E-07	5	NA	NA
BP	embryo development	1.72E-06	27	1.25E-06	22	1.00E+00	5
BP	single-organism developmental process	1.88E-06	72	1.46E-08	57	1.00E+00	15
BP	cellular component morphogenesis	2.43E-06	30	7.05E-08	26	1.00E+00	4
BP	developmental process	2.69E-06	72	2.04E-08	57	1.00E+00	15
BP	animal organ development	3.98E-06	50	3.51E-09	43	1.00E+00	7
BP	cell morphogenesis	8.32E-06	28	5.29E-07	24	1.00E+00	4
BP	columnar/cuboidal epithelial cell development	8.72E-06	8	3.83E-07	8	NA	NA

BP	retina homeostasis	1.01E-05	8	4.44E-07	8	NA	NA
BP	epithelial cell differentiation	1.42E-05	19	1.12E-08	19	NA	NA
BP	neuromuscular process controlling balance	1.53E-05	8	6.80E-07	8	NA	NA
BP	tissue homeostasis	1.78E-05	12	2.83E-06	11	1.00E+00	1
BP	cell projection organization	1.99E-05	28	1.77E-04	21	1.00E+00	7
BP	animal organ morphogenesis	2.23E-05	24	1.76E-07	22	1.00E+00	2
BP	detection of mechanical stimulus	2.29E-05	7	1.51E-06	7	NA	NA
BP	neuron development	3.76E-05	24	3.85E-04	18	1.00E+00	6
BP	system development	4.22E-05	57	6.36E-07	46	1.00E+00	11
BP	embryonic morphogenesis	4.78E-05	19	4.15E-08	19	NA	NA
BP	detection of mechanical stimulus involved in sensory perception	4.95E-05	6	4.91E-06	6	NA	NA
BP	neuron differentiation	4.96E-05	27	1.18E-04	21	1.00E+00	6
BP	multicellular organism development	5.51E-05	61	2.32E-06	48	1.00E+00	13
BP	epidermal cell differentiation	9.00E-05	11	1.34E-06	11	NA	NA
BP	columnar/cuboidal epithelial cell differentiation	1.25E-04	9	3.95E-06	9	NA	NA
BP	multicellular organismal homeostasis	3.45E-04	13	3.01E-05	12	1.00E+00	1
BP	photoreceptor cell maintenance	3.67E-04	6	3.72E-05	6	NA	NA
BP	generation of neurons	3.80E-04	27	6.34E-04	21	1.00E+00	6
BP	cellular developmental process	5.28E-04	53	2.40E-06	44	1.00E+00	9
BP	homeostatic process	1.10E-03	28	4.19E-03	21	1.00E+00	7
BP	tissue development	1.45E-03	30	1.42E-06	28	1.00E+00	2
BP	neuromuscular process	1.49E-03	8	7.27E-05	8	NA	NA
BP	neurogenesis	1.74E-03	27	2.25E-03	21	1.00E+00	6
BP	nervous system development	2.12E-03	33	3.79E-03	25	1.00E+00	8
BP	cell morphogenesis involved in differentiation	2.89E-03	18	2.28E-04	16	1.00E+00	2
BP	anatomical structure homeostasis	2.99E-03	12	3.72E-04	11	1.00E+00	1
BP	epidermis development	4.86E-03	11	8.75E-05	11	NA	NA
BP	localization	6.05E-03	61	1.00E+00	38	1.00E+00	23
BP	cell differentiation	9.42E-03	47	3.48E-04	38	1.00E+00	9
BP	sensory perception of light stimulus	9.62E-03	8	4.99E-04	8	NA	NA
BP	detection of abiotic stimulus	1.09E-02	7	8.09E-04	7	NA	NA
BP	detection of external stimulus	1.17E-02	7	8.67E-04	7	NA	NA
BP	epithelium development	1.18E-02	21	1.00E-05	21	NA	NA
BP	cellular component organization	1.33E-02	61	2.31E-02	44	1.00E+00	17
BP	response to mechanical stimulus	1.66E-02	9	6.27E-04	9	NA	NA
BP	establishment of localization	2.00E-02	51	1.00E+00	31	1.00E+00	20
BP	cochlea development	2.22E-02	5	3.49E-03	5	NA	NA
BP	cellular component organization or biogenesis	3.42E-02	61	4.95E-02	44	1.00E+00	17
BP	cellular cation homeostasis	4.08E-02	13	1.23E-01	10	1.00E+00	3
CC	stereocilium	1.10E-40	24	2.27E-45	24	NA	NA
CC	stereocilium bundle	5.43E-40	25	6.78E-45	25	NA	NA
CC	cluster of actin-based cell projections	4.28E-31	29	8.00E-37	29	NA	NA
CC	actin-based cell projection	3.53E-24	26	3.50E-29	26	NA	NA
CC	neuron part	3.11E-17	44	3.41E-14	33	2.10E-01	11
CC	stereocilium tip	1.31E-12	8	5.26E-14	8	NA	NA
CC	cell projection	8.24E-12	44	8.32E-11	34	1.00E+00	10

CC	plasma membrane region	4.97E-08	27	5.99E-08	22	1.00E+00	5
CC	cell projection part	3.32E-07	26	2.11E-05	19	1.00E+00	7
CC	apical part of cell	4.01E-07	17	1.05E-06	14	1.00E+00	3
CC	synapse	8.24E-05	20	2.81E-02	13	1.00E+00	7
CC	actin cytoskeleton	3.75E-04	15	9.78E-04	12	1.00E+00	3
CC	myosin complex	4.45E-04	7	8.97E-04	6	1.00E+00	1
CC	apical plasma membrane	6.90E-04	12	9.45E-04	10	1.00E+00	2
CC	nonmotile primary cilium	8.42E-04	9	2.81E-05	9	NA	NA
CC	cell junction	1.65E-03	24	1.69E-03	19	1.00E+00	5
CC	plasma membrane part	1.83E-03	36	1.10E-03	28	1.00E+00	8
CC	microvillus	2.00E-03	7	1.42E-04	7	NA	NA
CC	cell-cell junction	6.62E-03	13	2.88E-02	10	1.00E+00	3
CC	primary cilium	8.31E-03	9	3.03E-04	9	NA	NA
CC	stereocilia coupling link	1.25E-02	3	4.29E-03	3	NA	NA
CC	cell periphery	1.27E-02	62	1.02E-01	43	1.00E+00	19
CC	organelle	1.29E-02	109	1.00E+00	71	1.00E+00	38
CC	photoreceptor inner segment	1.74E-02	5	2.73E-03	5	NA	NA
CC	brush border	1.94E-02	7	1.47E-03	7	NA	NA
CC	kinocilium	2.17E-02	3	7.47E-03	3	NA	NA
CC	plasma membrane	2.97E-02	60	1.43E-01	42	1.00E+00	18
MF	actin binding	1.66E-06	17	3.40E-05	13	1.00E+00	4
MF	actin filament binding	2.15E-04	9	2.38E-03	7	1.00E+00	2
MF	cytoskeletal protein binding	2.24E-04	21	6.52E-03	15	1.00E+00	6
MF	actin-dependent ATPase activity	2.02E-03	4	4.59E-04	4	NA	NA
MF	calmodulin binding	2.15E-03	9	1.63E-01	6	1.00E+00	3
MF	protein binding	3.14E-03	101	1.88E-01	67	1.00E+00	34
MF	motor activity	9.62E-03	8	9.12E-02	6	1.00E+00	2
MF	microfilament motor activity	1.21E-02	4	2.78E-03	4	NA	NA

NA: No data available, BP: Biological Process, CC: Cellular Component, MF: Molecular Function
